# Supplementary material for: Function of crzA in Fungal Development and Aflatoxin Production in Aspergillus flavus
Source: Toxins (Basel). 2019 Sep 27;11(10):567. doi: 10.3390/toxins11100567 (PMC6832762; doi:10.3390/toxins11100567)
Supplement: Supplementary file 1 [file toxins-11-00567-s001.pdf]

Supplementary Materials: Function of *crzA* in Fungal Development and Aflatoxin Production in *Aspergillus flavus*

Su-Yeon Lim, Ye-Eun Son, Dong-Hyun Lee, Tae-Jin Eom, Min-Ju Kim and Hee-Soo Park

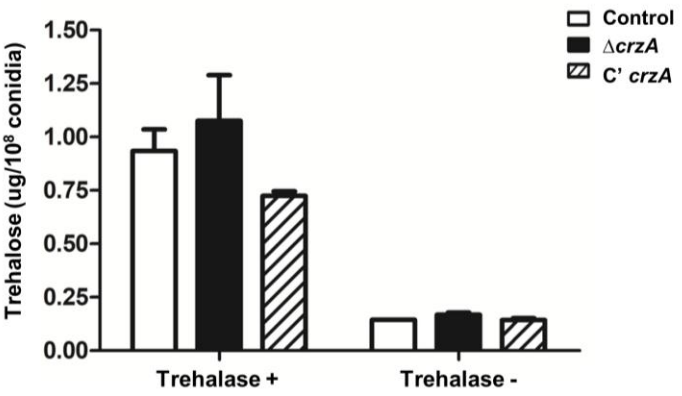

Figure S1. The amount of trehalose in Δ*crzA* mutant conidia. Amount of trehalose per 10^8 conidia from two-day plate culture of control (TTJ6.1), Δ*crzA* (TDH1.1) and C′*crzA* (TTJ9.1). The trehalose assay was performed as previously described (Eom et al., 2018). WT and mutants strains were grown on MMYE for 48 hours. After 2 days, conidia were collected from the plates. Conidia (10^8) were suspended in 200 µl of ddH_2O and incubated for 20 min at 95 °C. The supernatant was mixed with 0.2 M sodium citrate (pH 5.5) and incubated with or without trehalase for 8 h at 37°C. After incubation, the amount of glucose was assayed with a Glucose Assay Kit (Sigma) following the manufacturer’s instruction.

Eom, T. J., H. Moon, J. H. Yu and H. S. Park, 2018 Characterization of the velvet regulators in Aspergillus flavus. J Microbiol 56: 893–901.

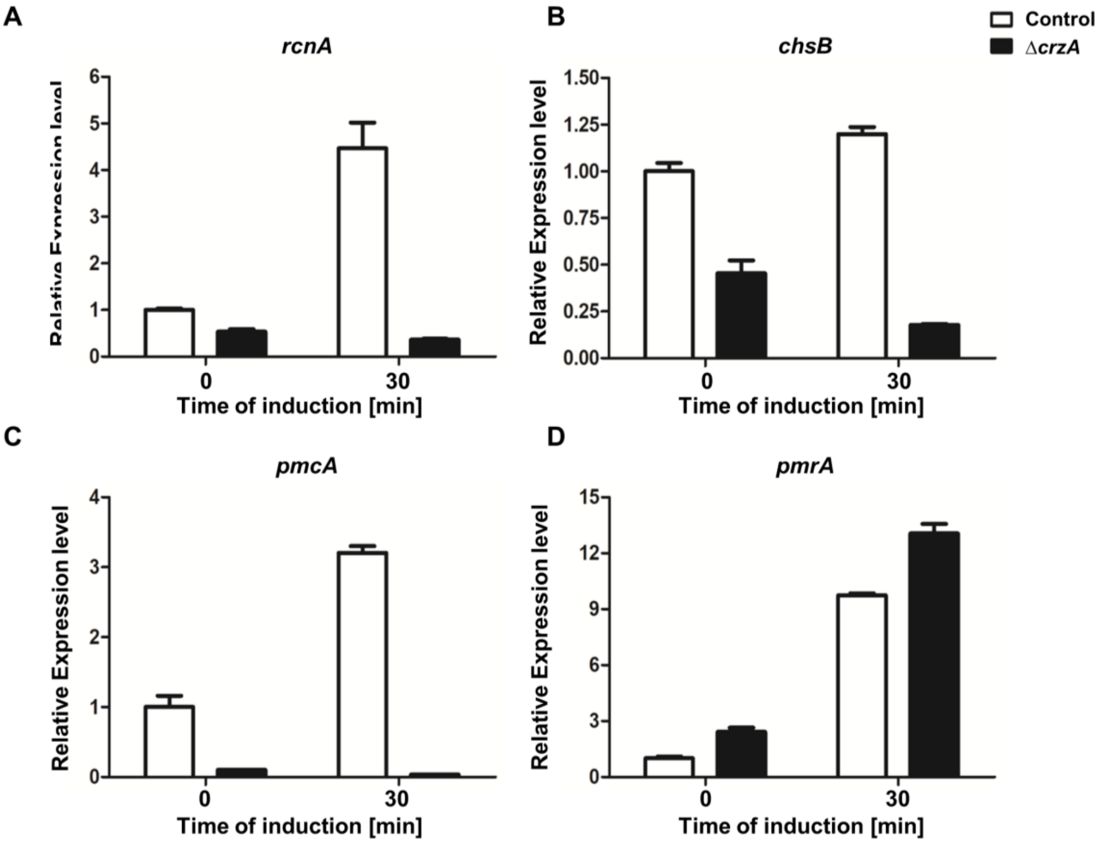

Figure S2 Expression of putative CrzA target genes. mRNA expression of *rcnA* (A), *pmcA*(B), *chsB*(C), and *pmrA*(D) in control (TTJ6.1) and Δ*crzA* (TDH1.1) mutant strains during exposure to high-calcium concentration. Conidia (10^6 conidia/ml) of control (TTJ6.1) and Δ*crzA* (TDH1.1) mutant strains were inoculated into liquid MMYE media and incubated for 18 hr at 37°C. After incubation, mycelia were shifted into fresh MMYE medium with 200 mM $CaCl_2$ for 30 min. RNA was extracted from the indicated strains and the mRNA expression of each gene was assessed by real-time PCR. Error bars depict the standard error of the mean for three independent experiments.

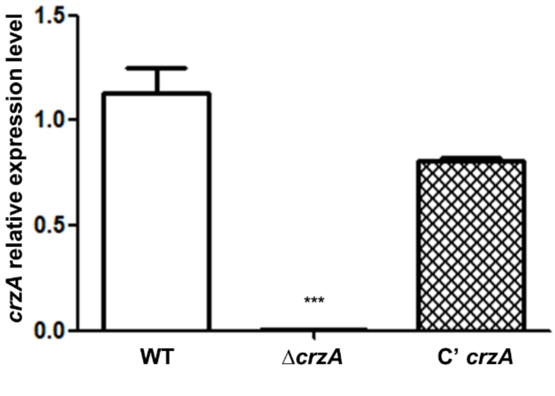

Figure S3 Verification of Δ*crzA* mutants by qRT-PCR. qRT-PCR analysis of *crzA* mRNA levels in WT, Δ*crzA* and C'*crzA* strains. The actin gene was used for an endogenous control. The error bars indicates the standard errors of the triplicate. (WT vs Δ*crzA*, *** $p \leq 0.001$; Δ*crzA* vs C'*crzA*, *** $p \leq 0.001$)

Table S1. Oligonucleotides used for Figure S2

**Name**	**Sequence (5′ → 3′)**	**Purpose**
OHS812	GAAATGGACGAGGTGCTTCC	*AflpmcA* F_RT
OHS813	GTTACCGCCACAGTTTCTCC	*AflpmcA* R_RT
OHS814	GAGCCATTTGAGGTGCACAA	*AflpmrA* F_RT
OHS815	CATGCACACGCGAAAGTCTA	*AflpmrA* R_RT
OHS818	ATTCCGGAGTTCATCTCGCA	*AflchsB* F_RT
OHS819	AGAATCTTTCGTGGCAGGGA	*AflchsB* R_RT
OHS933	GTCAATTCCTGCCTCTCCCT	*AflrcnA* F_RT
OHS934	TGTTGGCTTGGTTGGTTGAG	*AflrcnA* R_RT
